# Kinematic assessment of hip movement when retrieving an object from the floor

**DOI:** 10.1186/1749-799X-6-11

**Published:** 2011-02-21

**Authors:** Raheel Shariff, Sunil Panchani, John D Moorehead, Simon J Scott

**Affiliations:** 1 Orthopaedic Research Unit, University Hospital Aintree, Liverpool, L9 7AL, UK

## Abstract

**Background:**

Activities that require extreme hip movement can dislocate hip implants in the early post operative phase. One such activity is retrieving an object from the floor. The aim of this study was to assess hip movement using four different techniques to accomplish this task. This assessment would identify the techniques least likely to cause a hip dislocation.

**Methods:**

An electromagnetic tracker was used to measure the movement of 50 hips in 25 normal subjects. Sensors were attached over the iliac crest and the mid-shaft of the lateral thigh. Data was then collected for 3 repetitions of each of the following retrieval techniques:-

1. Flexing forward to pick up an object between the feet.

2. Flexing to pick up an object lateral to the foot.

3. Squatting to pick up an object between the feet.

4. Kneeling on one knee to pick up beside the knee.

**Results:**

Kneeling required a mean movement of 30.4 degree(s) flexion and 7.2 degree(s) external rotation. This was significantly less than all the other techniques (paired t-test, P << 0.001). Squatting required 87.4 degree(s) flexion and 10.1 degree(s) internal rotation.

**Conclusion:**

The study showed that squatting had the most flexion and internal rotation, whereas kneeling has the least flexion. Thus, to minimise the dislocation risk when retrieving an object from the floor, kneeling should be adopted and squatting should be avoided.

## Background

Total Hip replacements significantly improve the quality of life indices, with most patients returning to normal activities within 6 weeks [1]. However, 2 to 11% [2] of patients experience a post-operative dislocation. An initial dislocation may lead to recurrent dislocations causing a significant burden to the patient, surgeon and the health service. Factors predisposing to dislocation include patient compliance, implant positioning, elderly age, excessive alcohol and revision surgery [3,4].

After surgery many patients expect a rapid return to their normal activities of daily living (ADL's). However, some of these activities require hip movement [5] that increase the risk of joint dislocation. Thus, clinicians usually advise patients to restrict these movements in the early post operation phase [6]. The type of movement depends upon the surgical approach used for the hip implant. If a posterior approach was used then the patient should avoid excessive flexion and internal rotation [7]. If an anterior approach was used the patient should avoid excessive extension and external rotation [7].

Bending to pick up an object from the floor is an ADL that flexes the hip and poses a risk to posteriorly implanted joints. The aim of this study was to assess hip movement in normal subjects, using four different techniques to accomplish this task. These techniques could then be compared to see which one minimises the risk of dislocation in patients with a total hip replacement. We did not study patients with a total hip replacement in situ, as the 4 techniques used may theoretically put them at risk of a dislocation.

Previous studies have investigated hip movement during forward flexion [8,9] and squatting [5]. However, we believe our study is the first to compare various techniques for retrieving an object from the floor.

## Materials and methods

Study Design: Prospective study.

Ethical Committee Statement:

We state that our study has been approved by the Regional ethics committee and therefore has been performed in accordance with the ethical standards in the 1964 Declaration of Helsinki. All subjects gave their informed consent before their inclusion in the study.

### Power Calculation

To achieve a 5% significance level at a power of 0.80, assuming a medium effect size and a repeated measures design, it was calculated that a sample size of 24 would be required to obtain a statistical difference between the four techniques. A Student paired t-test was used.

### Subjects

Following regional ethics committee approval a total of 25 healthy volunteers were recruited for this prospective study. Strict inclusion and exclusion criteria were adhered to for recruitment as documented below.

#### Inclusion criteria

Individuals > 18 years of age

Mobilising without a walking aid

#### Exclusion criteria

History of Developmental dysplasia of the hip

History of trauma/fractures to hips or spine

Adduction contractures to hips

History of surgery to hips or spine

History of low back pain

### Instrumentation

A Polhemus Fastrak™magnetic tracking system was used to measure hip movement. Previous studies [10,9] have shown this tracker produces accurate and reproducible kinematic measurements.

The tracker consists of a 3- dimensional magnetic source and small 3- dimensional magnetic sensors connected to a computer. The source generated a small magnetic field which was detected by the sensors. One sensor was attached around the femur over the lateral aspect of the mid thigh. A second sensor was attached over the pelvis by the iliac crest. Each sensor was attached with a Velcro strap. It was then firmly secured to the skin with adhesive tape. As the sensors move through the source field they output position (X, Y and Z) and orientation (Yaw, Pitch & Roll) information to the computer at 12 Hz, with an accuracy of 0.15 degree.

The study was approved by the Regional Cheshire ethics committee and therefore had been performed in accordance with the ethical standards in the 1964 Declaration of Helsinki. All subjects gave their informed consent before their inclusion in the study.

### Procedure

The subjects were asked to stand upright with their arms by the sides. The position of the hip joint was recorded in this position and used as the reference for the subsequent movements. Hip movement was then recorded using four different techniques to retrieve a roll of tape from the floor. These techniques were:-

1. Flexing forward to pick up an object between the feet (Technique 1 - *Between*)

2. Flexing to pick up an object on the lateral side of the foot (Technique 2 - *Side*)

3. Squatting to pick up an object between the feet (Technique 3 - *Squat*)

4. Kneeling on one knee to pick up beside the knee. (Technique 4 - *Kneel*)

**Figure 1:**
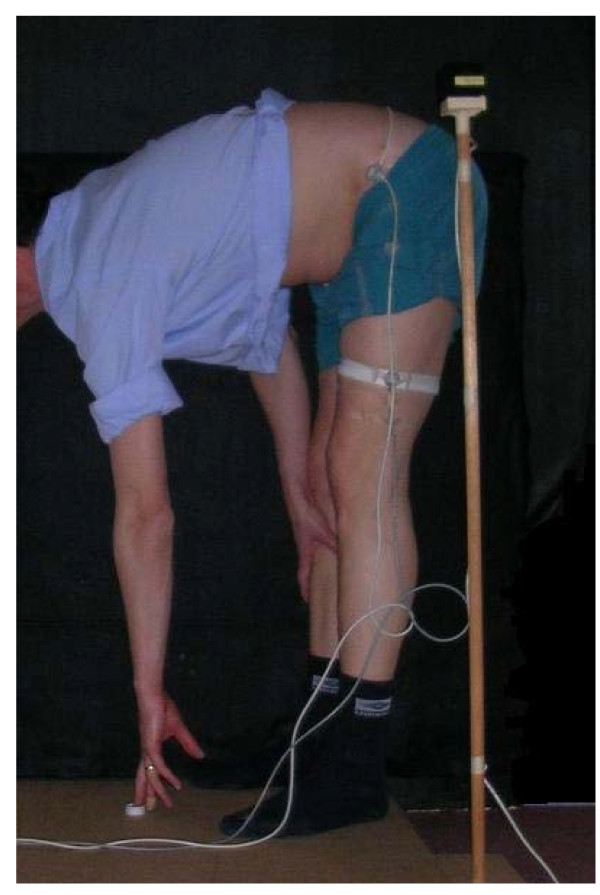
**Pick up between feet**.

**Figure 2:**
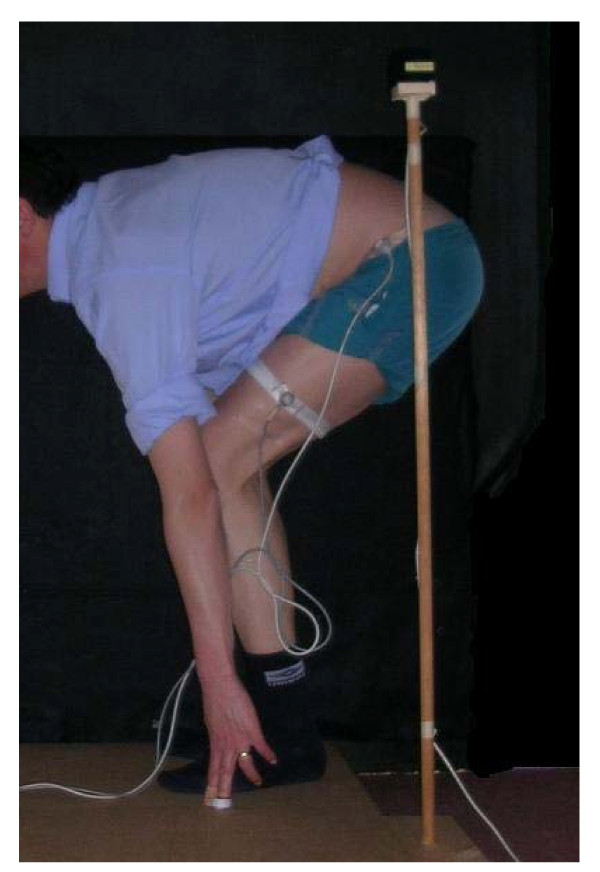
**Side pick up**.

**Figure 3:**
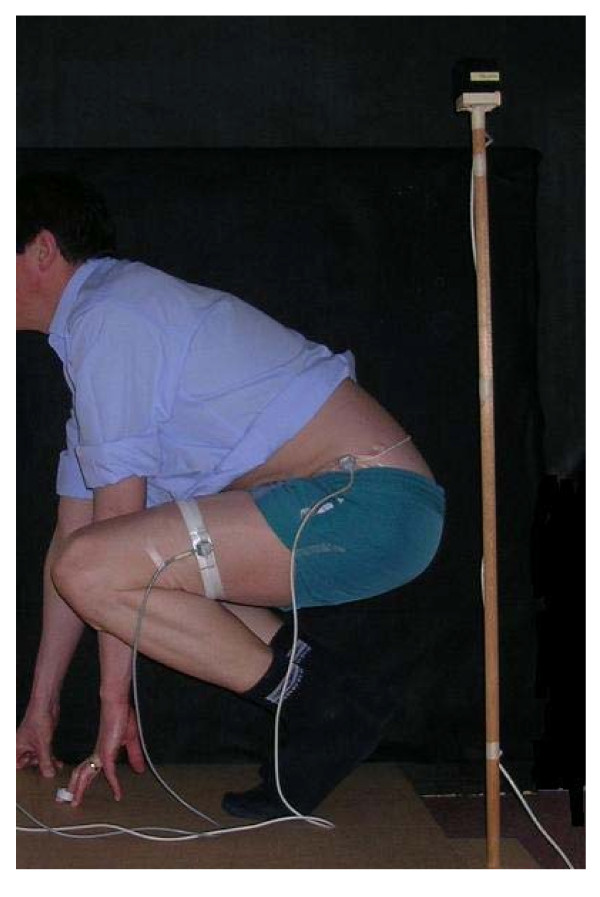
**Squat pick up**.

**Figure 4:**
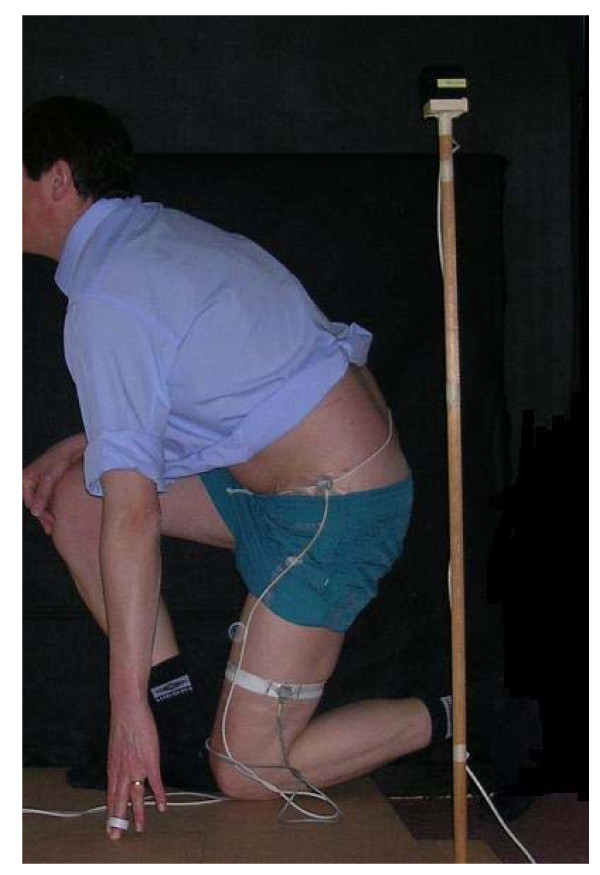
**Kneel pick up**.

The techniques are illustrated in Figures 1, 2, 3 and 4.

Foot placement and the placement of the object were standardized by marked reference points in the lab. Three continuous cycles were recorded for each technique in order to examine the repeatability of the movement. Sensors were not removed during the recording for each side. However they were checked in between each set of movements to ensure they were firmly attached. The procedure was then carried out on the contra lateral hip.

**Figure 5:**
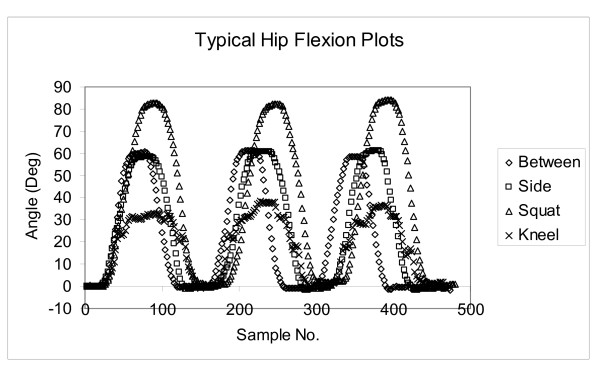
**Flexion plots for a typical subject using each of the retrieval techniques**.

Typical "Flexion - Sample Number" plots are shown in Figure 5. The plots show the hip flexions for one of the subjects retrieving an object from the floor using each of the techniques. The 3 cycles are shown, and they demonstrate good reproducibility for this subject. The Y data shows the amount of hip flexion required, and the × data shows the sample number, indicating time.

### Kinematic analysis

Hip flexion, extension, internal rotation and external rotation were analyzed for each of the four techniques. The techniques were then compared to see which one had maximal hip movements. Comparison was also made between the left and right sides.

## Results

**Figure 6:**
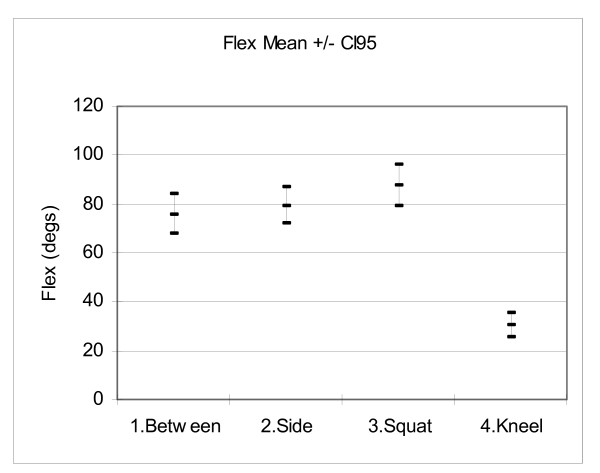
**Comparison of flexion**.

**Figure 7:**
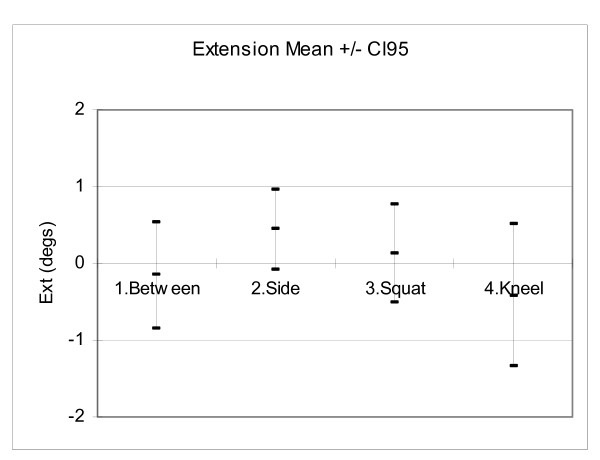
**Comparison of extension**.

**Figure 8:**
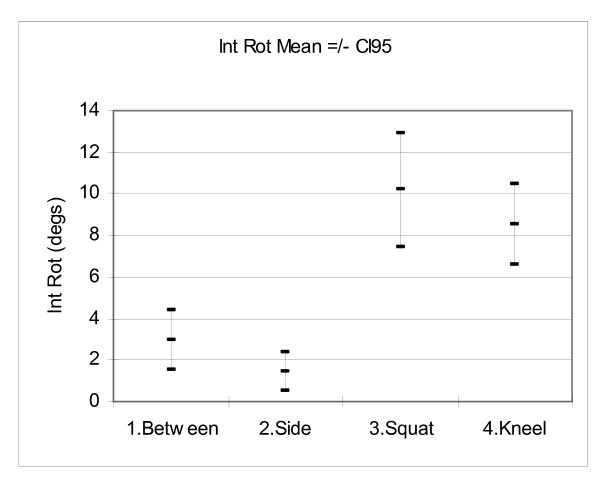
**Comparison of internal rotation**.

**Figure 9:**
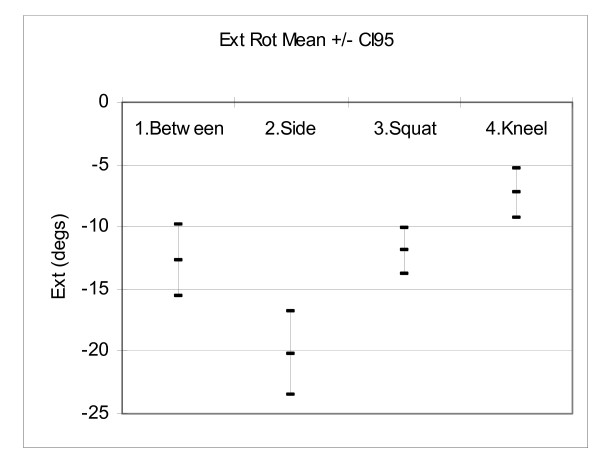
**Comparison of external rotation**.

Figures 6, 7, 8 and 9 show the mean hip flexion, extension, internal rotation and external rotation for all 50 hips (25 pairs), performing each of the retrieval techniques. These plots also show the 95% confidence intervals (CI95). Where there is no overlap between the CI95 bars, there is a significant difference in movement. Where there is an overlap, a paired t-test is required to determine significance.

Figure 6: When picking an object up between the feet, the mean flexion on the left side was 79.8. degree(s) On the right side it was 71.8 degree(s). This was the only movement that demonstrated a left/right difference, with a paired t-test P = 0.039. All other movements and techniques showed no left/right differences. Comparing flexion for each of the retrieval techniques, there was no significant difference between technique 1 (Between) and 2 (Side), paired t-test P = 0.08. Comparisons between techniques 1 & 3, 1 & 4, 2 & 3, 2 & 4 and 3 & 4 showed significant differences with P < 0.05.

Figure 7: For extension there was no significant left/right difference for each technique. There was no significant extension difference between any of the groups (P > 0.05).

Figure 8: For internal rotation there was no significant left/right difference for each technique. There was no significant internal rotation difference between techniques 1 & 2 and 3 & 4. There were significant differences between 1 & 3, 1 & 4, 2 & 3 and 2 & 4 (P < 0.05).

Figure 9: For external rotation there was no significant left/right difference for each technique. There was no significant external rotation difference between techniques 1 & 3. There were significant differences between techniques 1 & 2, 1 & 4, 2 & 3, 2 & 4 and 3 & 4 (P < 0.05).

## Discussion

### Flexion

Figure 6 shows the mean maximum hip flexion results. Technique 1 (Between) was the only technique to show a left/right difference, with P = 0.039. When the left hand was used there was 79.8 degree(s) of left hip flexion, when the right hand was used there was 71.8 degree(s) of right hip flexion. The reason for the reduced hip flexion on the right side is unclear, but it may be related to hand dominance, scapula protraction and trunk rotation. For this movement the P value (0.039) was approaching borderline significance. The other techniques had no significant left/right difference. To simplify a comparison between techniques, Figure 6 shows the pooled left/right data for each technique. Examination of the plots shows that technique 4 (kneel) had the least flexion (30.4 degree(s)). This was followed by technique 1 (between) with 75.8 degree(s), technique 2 (side) with 79.2 degree(s) and technique 3 (squat) with 87.5 degree(s).

The mean peak flexion for technique 1 is similar to readings found in other studies which looked at forward flexion [8,11]. Extreme forward flexion is a movement from which patients in the early post-operative period are protected. This is done mainly by technique modification education conducted by physiotherapists during rehabilitation. This minimises femoral neck impingement on the acetabular lip, and thus dislocation [12]. Khan et al conducted a multi-centre study and found that 31 out of a total of 142 dislocations occurred due to forward flexion (22%) [3].

In initial visual observations it appeared that technique 1 (between) had the most flexion. However, a full analysis of the measured data showned that technique 3 (squat) has the most flexion (87.5 degree(s)). This surprising result may be explained by the coupled movement of the lumbar spine and hip joint aiding flexion in technique 1 [8]. Kneeling had the least flexion and therefore poses the least risk of hip dislocation.

### Extension

Figure 7 shows the mean maximum hip extension results for each technique. It shows that none of the techniques required significant extension past the neutral reference position. Technique 4 (kneel) had the most extension (0.4 degree(s)). Extension is a risk factor for hips implanted with an anterior approach [7]. These small extensions should not pose a significant dislocation risk to patients.

### Internal Rotation

Figure 8 shows the mean maximum internal rotation results. Techniques 2 (side) and 1 (between) had the least internal rotation, with values of 1.4 degree(s) and 2.9 degree(s) respectively. Techniques 4 (kneel) and 3 (squat) had the most internal rotation, with values of 8.5 degree(s) and 10.2 degree(s) respectively.

Technique 3 (squat), also had the most flexion. Flexion coupled with internal rotation can predispose a hip to dislocation when it has been implanted with a posterior approach [7]. Our results indicate that this technique should be avoided to minimise the dislocation risk.

Technique 4 (kneel) had the least flexion. Although it's internal rotation was greater than techniques 1 and 2, its minimal flexion makes it safer than the other three techniques.

### External Rotation

Figure 9 shows the mean maximum external rotation results. Technique 4 (kneel) had the least external rotation with 7.3 degree(s). Techniques 3 (squat) and 1 (between) had external rotations of 11.9 degree(s) and 12.7, degree(s) respectively. Technique 2 (side) had the most external rotation, with 20.1 degree(s). External rotation mainly poses a risk to patients with a hip implanted via an anterior approach. The greatest risk occurs when the external rotation is coupled with hip extension. As there was very little extension past neutral for any of the techniques, it is unlikely that the recorded external rotations will pose a significant dislocation risk.

### Spinal movement

Each of the retrieval techniques also required spinal movement coupled to the hip movement. Esola et al described the pattern of motion during forward flexion by calculating lumbar to hip flexion ratios [8]. For normal subjects they found that in early flexion (0-30 degree(s)), the lumbar-hip flexion ratio was 1.59. In mid flexion (30-60 degree(s)) the ratio was 1.06. In late flexion (60-90 degree(s)) the ratio was 0.49. Thus in early flexion, the lumbar spine contributed more than the hip. In late flexion, the hip contributed more than the lumbar spine. They concluded that both the lumbar spine and hip joint contribute to bending forward movement, but the lumbar spine mainly contributes to the early part of this movement. In patients with low back pain this ratio was increased. As patients with back pain were excluded from our study, we assume that the lumbar spine played its normal role in the bend forward manoeuvre.

### Other high risk activities

Retreiving an object from the floor in the early post operative period can be considered a high risk activity given the amount of hip movement involved. Meek et al looked at the epidemiology of hip dislocations and recommended that high risk activities should be avoided for at least one year post operative [6]. Hip dislocation has been classified as a result of patient position, soft tissue imbalance and component malposition [13]. Nadzadi et al studied the kinematics of activities of daily living which pre-dispose to dislocation. They found that standing from a low seated position had the highest risk of posterior dislocation. This was followed by standing from a seated position at normal height [14]. In another study Hemmerich et al found that high ranges of hip movements were not provided by most currently available prosthesis in the market.

## Conclusion

The aim of this study was to study and suggest the postural method of retrieving an object from the floor for patients undergoing total hip replacement surgery.

The present study provides useful information on the normal kinematics of hip joint movements when retrieving an object from the floor. The technique most at risk of dislocation is squatting. The technique with the least risk of dislocation is kneeling. From these results it is recommend that kneeling is adopted for post operative rehabilitation and mobilisation protocols, following total hip arthroplasty.

## Competing interests

The authors state that they have no financial relationship with the organization that sponsored the research. We also state that we have full control of all primary data and that we agree to allow the journal to review our data if requested. 'The author(s) declare that they have no competing interests'.

We have not received reimbursements, fees, funding, or salary from an organization that may in any way gain or lose financially from the publication of this manuscript, either now or in the future.

The authors do not hold any stocks or shares in an organization that may in any way gain or lose financially from the publication of this manuscript, either now or in the future

The authors are not currently applying for any patents relating to the content of the manuscript. Have not received reimbursements, fees, funding, or salary from an organization that holds or has applied for patents relating to the content of the manuscript

Non-financial competing interests

The authors do not have any non-financial competing interests (political, personal, religious, ideological, academic, intellectual, commercial or any other) to declare in relation to this manuscript.

## Consent

Written informed consent was obtained from the patient for publication of this article and accompanying images. A copy of the written consent is available for review by the Editor-in-Chief of this journal

## Authors' contributions

The authors have all read and agree with the manuscript. RS drafted the protocol, sought ethical approval, collected data, helped in analysis and drafted the manuscript. SP helped collect data, analyse and draft the manuscript. JM participated in drafting the protocol, collecting data, analysis, statistics and drafting the manuscript. SJS helped draft the protocol and prepare the manuscript.
